# MixHMM: Inferring Copy Number Variation and Allelic Imbalance Using SNP Arrays and Tumor Samples Mixed with Stromal Cells

**DOI:** 10.1371/journal.pone.0010909

**Published:** 2010-06-01

**Authors:** Zongzhi Liu, Ao Li, Vincent Schulz, Min Chen, David Tuck

**Affiliations:** 1 Department of Pathology, Yale University School of Medicine, New Haven, Connecticut, United States of America; 2 Department of Pediatrics, Yale University School of Medicine, New Haven, Connecticut, United States of America; 3 Department of Epidemiology and Public Health, Yale University School of Medicine, New Haven, Connecticut, United States of America; Memorial Sloan Kettering Cancer Center, United States of America

## Abstract

**Background:**

Genotyping platforms such as single nucleotide polymorphism (SNP) arrays are powerful tools to study genomic aberrations in cancer samples. Allele specific information from SNP arrays provides valuable information for interpreting copy number variation (CNV) and allelic imbalance including loss-of-heterozygosity (LOH) beyond that obtained from the total DNA signal available from array comparative genomic hybridization (aCGH) platforms. Several algorithms based on hidden Markov models (HMMs) have been designed to detect copy number changes and copy-neutral LOH making use of the allele information on SNP arrays. However heterogeneity in clinical samples, due to stromal contamination and somatic alterations, complicates analysis and interpretation of these data.

**Methods:**

We have developed MixHMM, a novel hidden Markov model using hidden states based on chromosomal structural aberrations. MixHMM allows CNV detection for copy numbers up to 7 and allows more complete and accurate description of other forms of allelic imbalance, such as increased copy number LOH or imbalanced amplifications. MixHMM also incorporates a novel sample mixing model that allows detection of tumor CNV events in heterogeneous tumor samples, where cancer cells are mixed with a proportion of stromal cells.

**Conclusions:**

We validate MixHMM and demonstrate its advantages with simulated samples, clinical tumor samples and a dilution series of mixed samples. We have shown that the CNVs of cancer cells in a tumor sample contaminated with up to 80% of stromal cells can be detected accurately using Illumina BeadChip and MixHMM.

**Availability:**

The MixHMM is available as a Python package provided with some other useful tools at http://genecube.med.yale.edu:8080/MixHMM.

## Introduction

Chromosomal structural abnormalities leading to copy number changes, including deletions and amplifications, are common in cancer and certain regions are commonly altered, suggesting their role in the pathogenesis of this disease [1], [2]. Copy number variation (CNV) in the germ line is increasingly recognized as contributing to developmental defects and susceptibility to diseases including cancer, similar to single nucleotide polymorphisms (SNP) [3], [4]. Copy number somatic alterations (CNA, also referred as CNV here after, as we use the same algorithm for detection) have been reported as an important factor leading to cancer [5]. Higher resolution detection of CNV contributes to the basic understanding of tumor progression and to the development of biomarkers for prediction of response to therapy [6]. Advances in the understanding of the relationships of CNV to basic genomic and epigenomic features of tumors make it important to extract as much information as possible from the data available.

The methods for identification of CNV have improved since the first low resolution cytogenetic and comparative genomic hybridization studies [7]. Array comparative genomic hybridization (aCGH) uses arrays of bacterial artificial chromosome, cDNA, or synthetic oligonucleotides to probe specific chromosomal regions for differences in copy number [8], [9]. The aCGH hybridization signal is segmented by chromosomal location [10], [11], and changes in intensity over a region reflect changes in copy number.

Compared to aCGH methods, whole genome genotyping arrays based on SNPs (such as the Illumina BeadArray) allow for combined copy number analysis and allelic imbalance analysis at high resolution [12]. Starting from the signal intensities of two SNP alleles, the Illumina platforms yield two transformed parameters after self normalization and comparison with reference normal samples: log R ratio (LRR) derives from the total signal intensity of both alleles and only depends on the copy number, while ‘B’ allele frequency (BAF) derives from allele signal intensity ratio and depends on the allele ratio (i.e. proportion of ‘B’ in a genotype composed of ‘A’s and/or ‘B’s). The values of LRR and BAF for each SNP can be plotted along the entire genome in the position order. A LRR plot of a diploid chromosomal region displays a band centered at 0, and a region with copy number changes will be reflected by an upward or downward shift of the band. A BAF plot of a sample which is either normal or contains balanced amplifications (both alleles are amplified to the same copy number) displays as a three-band pattern, with homozygous genotypes clustering at 0 or 1 and heterozygous genotype clustering at 0.5. A LOH region, representing the most imbalanced form of CNV, lack any heterozygous bands, while an allelic imbalanced region other than LOH will be reflected as a split of the heterozygous band in the BAF plot. In tumor samples, both alterations in copy number and ‘contamination’ of stromal cells (which are typically seen) can contribute to the more complex band patterns [12], [13].

Most approaches to analysis of whole genome genotyping arrays have used either segmentation or probabilistic approaches. A number of segmentation algorithms have been developed to combine BAF and the total DNA signal, generally by removing homozygous SNPs from the BAF and transforming the BAF of the remaining SNPs so they are independent of the specific allele, using some relationship to the normal heterozygous position of 0.5 [13], [14]. These methods require user defined or adaptively derived thresholds and the biological assumptions are usually unrealistic; for instance, Assie et al. [14] assumed that all the amplifications are three copy. Hidden Markov models (HMM) are elegant and powerful methods addressing the probabilistic approach. The model proposed by QuantiSNP [15] and adapted by PennCNV [16], was specifically devised to take advantage of the total DNA and allele specific data that is provided by genotyping platforms. They have provided valuable tools for the analysis of the homogeneous samples. However, they were not designed for the precise delineation of allelic imbalance (only copy-neutral LOH can be detected), nor to take into account the fact that tumor samples may frequently contain DNA that comes from a mixture of tumor and stromal cells. dChip and overunder are two algorithms which were designed to deal with tumor samples but do not handle admixtures with stromal cells [17], [18]. In a very recent publication, Sun et al. [19] have addressed the problem of stromal contamination, but the CNV assignment is inaccurate in tumor samples with a considerable proportion of normal stromal cells (see results).

Using a HMM with up to 20 states representing copy numbers from 0 through 7, we developed a novel computational framework (MixHMM) for detecting copy number and allelic imbalance accurately. By combining with a novel sample mixing model, we demonstrate that MixHMM can also detect the CNV states of tumor cells in a heterogeneous sample contaminated with normal cells (i.e. in a biopsy sample). The remainder of the paper is structured as follows. First, we present the underlying assumptions, the CNV states, our definition of allelic imbalance and the HMM. Second, we present the sample mixing model which allows us to detect copy number changes and allelic imbalance in mixed tumor samples. We then validate the algorithm on simulated data and illustrate the essential features. Next, we show the results of dilution series in which tumor DNA is mixed with normal DNA. Finally, we demonstrate that the algorithm can be applied with either pure or mixed tumor samples from patients.

## Results

### The CNV states and Hidden Markov Model

**Figure 1:**
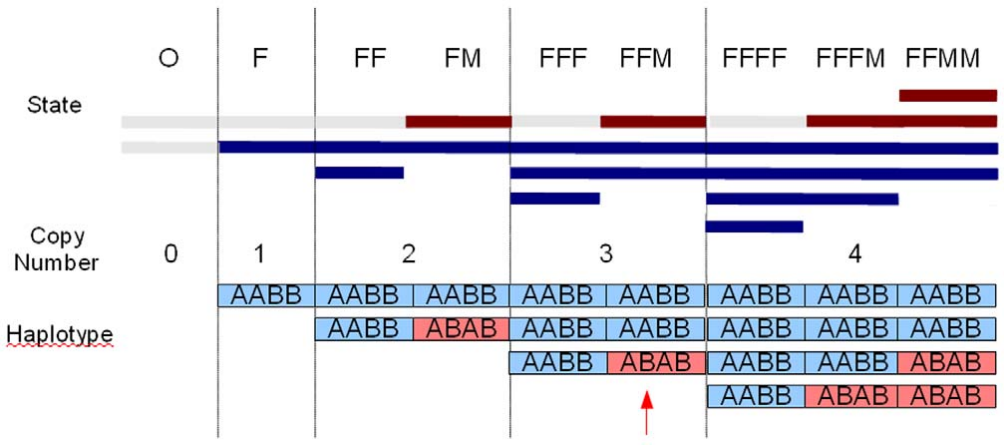
Chromosome instability events as CNV states for copy number up to four. All nine possible CNV states and genotypes with copy numbers up to 4 are presented here as a “pseudo chromosome”. (See Table 1 for an alternative representation of 20 states with copy numbers up to 7). All states are assumed to be derived from the underlying normal two copy state (‘FM’) which has regions from both chromosomes (‘F’ in blue, ‘M’ in red). The top track indicates the composition of each state based on the source chromosomes. The second track gives a graphical representation of the state composition along different regions. The third track gives the copy number for a region, from 0 to 4, which are separated by the vertical bars. The fourth track shows an example set of haplotypes making up the region (‘A’ and ‘B’ are the alternate alleles). There are up to four distinctive genotypes in each state, with each genotype for an individual SNP shown in a vertical column (for example, the SNP genotype indicated by the red arrow is ‘AAB’). In the homozygous deletion state (‘O’), both regions are deleted (labeled in gray). In the LOH states (labeled with only ‘F’s), one of the source chromosomes is deleted, while the other can be amplified one or more times. The normal state (‘FM’) has regions from both chromosomes. The remaining states harbor regions from both source chromosomes with one or both regions amplified. States such as ‘MM’, ‘FMM’, etc are not listed because they are not distinguishable from ‘FF’ and ‘FFM’ by genotying array data.

Copy number variation (CNV) events such as deletion and duplication/amplification can be detected from genotyping array data, which give BAF and LRR values for each SNP based on the signal intensities of both SNP alleles [12]. Figure 1 is a schematic representation of those CNV events up to copy number 4. We use ‘F’ and ‘M’ throughout to represent each of a pair of homologous chromosomes inherited from parents. We make the assumption that each CNV state originated from the underlying normal two copy state (‘FM’) with one or both chromosomes deleted or amplified. The upper part of Figure 1 demonstrates that there are nine distinctive CNV states from 0 to 4 copies. We always use fewer or an equal number of ‘M’s in a state name because a state like ‘FMM’ is not distinguishable from ‘FFM’ by genotyping array data. Each state defined as above is distinct from the other states based on the combination of its copy number (CN) and its allelic imbalance (AI).

In Table 1, we list all the possible CNV states for copy number up to seven. For a quantitative measurement, we define allelic imbalance of a CNV state as 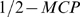, where MCP stands for the proportion of the minor copy allele (i.e. the proportion of ‘M’s in a state name in Figure 1 and Table 1). Thus, by definition, the allelic imbalance is a value between 0 and ½ (including borders). Allelic imbalance of the normal state (‘FM’) or a balanced amplification states (containing equal numbers of ‘F’ and ‘M’, such as ‘FFMM’) is 0; that of a LOH state (with only ‘F’s in name) is 1/2; that of an imbalanced amplification (in which both alleles are present in increased but unequal numbers, such as ‘FFM’) will be a value between 0 and 0.5 (for state ‘FFM’, 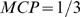 and allelic imbalance 
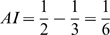
). Therefore, by using the CNV states defined above as the hidden states in the hidden Markov model (HMM), we can detect forms of allelic imbalance other than LOH, such as imbalanced amplification.

**Table 1:** CNV states and Genotypes.

CNV	Copy	Minor Copy	Genotype Classes			
state	number	Proportion	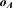	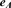	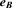	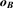
O	0	NA	-	-	-	-
F	1	0	A	A	B	B
FF	2	0	AA	AA	BB	BB
FM	2	1/2	AA	AB	AB	BB
FFF	3	0	AAA	AAA	BBB	BBB
FFM	3	1/3	AAA	AAB	ABB	BBB
FFFF	4	0	AAAA	AAAA	BBBB	BBBB
FFFM	4	1/4	AAAA	AAAB	ABBB	BBBB
FFMM	4	1/2	AAAA	AABB	AABB	BBBB
FFFFF	5	0	AAAAA	AAAAA	BBBBB	BBBBB
FFFFM	5	1/5	AAAAA	AAAAB	ABBBB	BBBBB
FFFMM	5	2/5	AAAAA	AAABB	AABBB	BBBBB
FFFFFF	6	0	AAAAAA	AAAAAA	BBBBBB	BBBBBB
FFFFFM	6	1/6	AAAAAA	AAAAAB	ABBBBB	BBBBBB
FFFFMM	6	1/3	AAAAAA	AAAABB	AABBBB	BBBBBB
FFFMMM	6	1/2	AAAAAA	AAABBB	AAABBB	BBBBBB
FFFFFFF	7	0	AAAAAAA	AAAAAAA	BBBBBBB	BBBBBBB
FFFFFFM	7	1/7	AAAAAAA	AAAAAAB	ABBBBBB	BBBBBBB
FFFFFMM	7	2/7	AAAAAAA	AAAAABB	AABBBBB	BBBBBBB
FFFFMMM	7	3/7	AAAAAAA	AAAABBB	AAABBBB	BBBBBBB

A CNV state is named using ‘O’ (for homozygous deletion) or a combination of ‘F’s and ‘M’s, with less or equal number of ‘M’s. Minor copy proportion (MCP) is the proportion of the number of ‘M’s in a state name. The four genotype classes are defined by their germline origination: 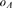,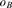 originate from germline homozygous genotypes ‘AA’ and ‘BB’, respectivly; 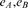 originate from germline heterozygous genotype ‘AB’.

A’ and ‘B’ to represent the two investigated alleles for each SNP, the bottom track in Figure 1 shows that each CNV state can include up to four different genotypes (each genotype should be read vertically), with each genotype corresponding to a characteristic horizontal band in a BAF plot for a homogeneous sample. Each LOH state has exactly two distinctive genotypes; each allele balanced state has three distinctive genotypes; and each imbalanced state other than LOH has four distinctive genotypes. As shown in Table 1, we classify genotypes of each state into four classes based on the original germline genotypes (‘AA’, ‘BB’, ‘AB’): derived from original ‘AA’ 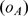, derived from original ‘BB’ 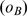, derived from original ‘AB’ with an equal number or more of ‘A’s compared to ‘B’s 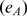, and derived from original ‘AB’ with equal number of or more of ‘B’s compared to ‘A’s 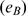. Let 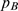 be the population frequency of ‘B’ allele at a SNP locus, then the probabilities of observing each genotype in a normal state (‘FM’) are 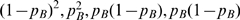 for genotypes 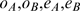 respectively.

Under the assumption stated in the first paragraph (all the CNV states originated from ‘FM’ states with only deletions and/or amplifications), we can deduce that the probability of observing each of the four genotype classes of a given SNP is exactly the same as that in ‘FM’ state. Therefore, we use Equ. 4 (see methods section for equations) to estimate the probability of observing a BAF value, given a CNV state and the BAF distribution of each of the four genotype classes; we use Equ. 3 to estimate the probability of observing an LRR value, given a CNV state and its LRR distribution. Under the same assumption, we can also deduce that, given that there is a state change between two adjacent SNPs, the state of the second SNP is independent of that of the first one. Therefore, we use Equ. 2 to estimate the state transition probabilities, given the prior probabilities of state changing. These estimates are subsequently used in the Viterbi algorithm to decode the hidden states of each SNP locus in each chromosome.

### Model update in tumor samples mixed with stromal DNA

To detect the CNV states in a non-homogeneous tumor sample ‘contaminated’ with a known proportion (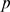) of the stromal cells (assumed to be in ‘FM’ state by default), we update the LRR and BAF normal distributions using separate mixing models.

**Figure 2:**
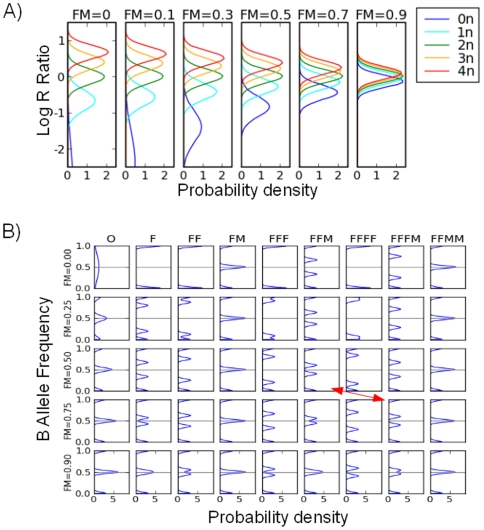
LRR distributions and BAF distributions in simulated mixed samples. A) Mixing of LRR. Each line represent a state of a certain copy number (color code on right) mixed with a proportion of normal ‘FM’ cells (proportion on top), with ‘FM = 0’ corresponding to a pure tumor sample. B) Mixing of BAF. Each subplot represent a certain CNV state (name on top) mixed with a proportion of ‘FM’ cells (proportion on left), with ‘FM = 0’ corresponding to a pure tumor sample.

We use Equ. 6 and Equ. 7 to calculate the LRR distributions for each CNV state mixed with normal state. Figure 2a shows the results for five different copy number states (0 to 4 copies) mixed with different proportions of normal (‘FM’) cells. When there is no normal tissue included (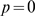), the LRR distributions are exactly the same as those of pure tumors. With the proportion of normal cells increasing, the mixed signals are more influenced by the normal DNA; thus, the LRR distributions (for both mean and variance) of all other copy number states start to shift toward the distribution of the diploid state (2n, the green line in Figure 2A). As a consequence, the power to discriminate different states decreases, especially for the states with a higher copy number.

Assuming that each CNV region in tumor cells is derived from the corresponding region in the mixed stromal cells, we can deduce that each compound ‘genotype’ in a mixed sample comes from the genotypes of the same class in tumor and normal DNA (see Table 1). For example, when a ‘FFM’ tumor state is mixed with the ‘FM’ normal state, the BAF distribution of the mixed 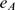 ‘genotype’ must come from a mixture of tumor 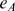 genotype (‘AAB’) and normal 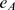 genotype (‘AB’). Thus the distribution probabilities for the four genotypes stated in Equ. 4 (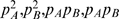) still applies.

However, the BAF normal distributions do change after mixing, and we use Equ. 9 and Equ. 10 and to estimate the BAF distribution of each of the four compound ‘genotypes’. Figure 2B shows the results from nine CNV states (as columns), representing copy number 0 through 4, mixed with four different proportions (as rows) of normal cells. As expected, the BAF distributions for the mixed samples for each state converge to the BAF distributions of ‘FM’ state as the proportion of the normal cells increases. Different kinds of states are affected in different ways. Specifically, the balanced states (‘FM’ and ‘FFMM’) stay the same and are not affected by the presence of normal cells. The homozygous deletion state (‘O’) approaches the normal state as normal cells are added to the mixture. For the LOH states (‘F’, ‘FF’, ‘FFF’, ‘FFFF’), two heterozygous bands emerge as the result of 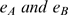 genotype mixing respectively. For example, 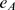 in ‘F’ state (‘A’) is mixed with 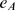 in ‘FM’ state (‘AB’). Imbalanced amplifications (‘FFM’, ‘FFFM’), which already have two heterozygous bands, also converge to the ‘FM’ state as the proportion of normal cells increases.

Thus, it is evident that the predictive power of MixHMM decreases with the increasing noise level in the data caused by ‘contamination’ of stromal cells. We will show below, however, that in both simulations and real tumor samples, MixHMM can reliably detect the CNV states up to seven copies in a sample mixed with up to 0.6 proportion of normal cells. We can also see from Figure 2 that the correct assignments of CNV states in tumor can be negatively influenced by inaccurate estimation of the proportion of cells in normal state. For example, ‘FFM’ mixed with a 0.5 proportion of ‘FM’ has an identical BAF distribution as that of ‘FFFM’ mixed with a 0.75 proportion of ‘FM’ (subplots indicated by arrows in Figure 2); and the mixed copy numbers are also identical, which is 2.5. Therefore if the proportion 0.5 is inaccurately estimated to be 0.75, the ‘FFM’ state is likely to be misassign ed to ‘FFFM’ state. Assuming that the mixed samples are composed of homogeneous pure tumor cells and homogeneous stromal cells, we use Equ. 11 to estimate the proportion of normal cells from the characteristic BAF value of the compound 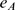 genotype for a given tumor CNV state, and we found that the CNV detection of MixHMM is pretty robust with proportion estimation.

### Evaluation with simulated data

To evaluate the performance of MixHMM, we simulated regions of all states with a 20-state model using the SNP positions and population ‘B’ allele frequencies (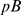) of the Illumina Human550K BeadChip (Illumina. http://www.illumina.com).

**Figure 3:**
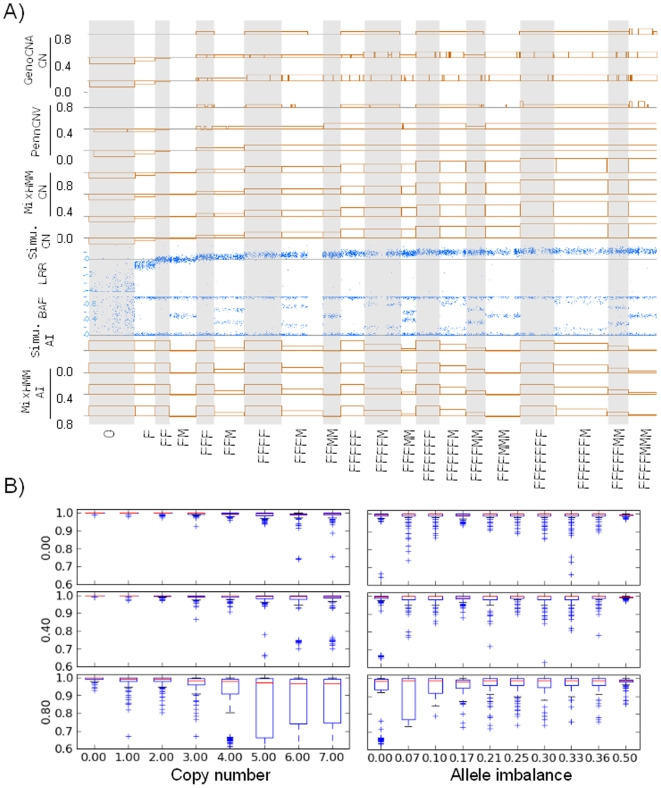
CNV detection from simulated data. A) Detection of copy number (CN) and allelic imbalance (AI) from simulation of pure tumor and mixed samples on Chromosome 1. Each of the 20 states are simulated to be a 300-SNP region. The numbers on the left side are proportions of ‘FM’ cells. The underlying truth simulated is depicted in the panels of ‘simu. CN’ and ‘simu. AI’. The BAF and LRR plots are of simulated pure tumor cells (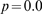). In the PennCNV and GenoCNA CN tracks, the copy number are from 0 to 4 with the baseline (gray) representing 2n, and flat box (the orange fragment) is copy neutral LOH. The results of MixHMM are separated to copy number and allelic imbalance. In the CN tracks, the baseline (gray) represents 2n, and the copy numbers range from 0n through 7n. In the AI tracks, the baseline represents 0, and it ranges from 0 through 0.5. B) Box plots of recovery rates of copy number and allelic imbalance detected using MixHMM from the simulation. The numbers on the left side are proportions of ‘FM’ cells. Values of each copy number/imbalance comes from the simulations of 220 regions with each region spaning 100 SNPs.

To evaluate the algorithm visually, we simulated each state (in the same order as in Table 1) as a 300-SNP region on chromosome 1. The results of CNV detection by MixHMM is shown in Figure 3A. We show that both copy number and allelic imbalance are detected accurately for all the 20 states in the simulated pure tumor sample (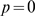). Also, the CNV detection in simulated tumor sample mixed with up to 80% normal cells are almost as accurate. With this simulation data, incorrect state assignments only occur in the bordering area between two adjacent regions with different CNV states, especially between regions with the same copy number, hence the same expected LRR value (data not shown). As homozygous genotypes exist in all the CNV states (see Table 1), and they have the same expected BAF values (0/1), when several SNPs of such genotypes are in the bordering region, it is not possible to draw an exactly correct border line.

To compare MixHMM with other detection algorithms, the results of PennCNV [16] and GenoCNA [19] for the same simulation data as above are shown on the top tracks of Figure 3A. They only detected the copy number from 0 through 4, and these detections become inaccurate in samples with a considerable proportion (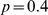,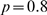) of normal cells. For example, the four copy (4n) regions and ‘FFFMMM’ (6n) regions tend to be misassigned as three copy (3n) when 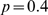, all the deletion regions and many amplicated regions have not been detected when 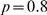.

To evaluate the CNV detection quantitatively, we simulated 20 states (with shuffled order and different chromosomal offset position) on every autosomal chromosomes. We have used different region lengths (50, 100, 200, 300 SNPs) in each simulation. We define recovery rate as the proportion of SNPs with detected value (copy number or allelic imbalance) exactly the same as the underlining true value. Figure 3B shows the recovery results from 100 simulations (220 duplications for each state) with 100-SNP CNV regions. We can see that the detection of copy number is less accurate when the proportion of normal cells is very high (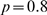), especially for regions with a high copy number (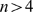). For states with high copy number, the differences of LRR values between states are smaller (also see Figure 2A), so CNV states with similar mixed BAF distributions are more likely to be confounded with each other (for example, ‘FFFFMM’ and ‘FFFFFMM’). The detection of allelic imbalance is also less accurate when 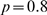, especially for regions with a small allelic imbalance. The BAF of these states look more like that of ‘FM’ state (also see Figure 2B), so the CNV states with similar mixed ‘copy numbers’ can be misassigned to each other (for example ‘FFFMMM’ and ‘FFFFMMM’). However, such misassignments are almost always between high copy number states, and usually do not pose a problem for CNV conclusions. We also found that even these trivial misassignments become less common with larger CNV regions (regions with more than 200 SNPs). The recovery results for 300-SNP regions are included in Figure S1.

### Evaluation with dilution series of Cancer Celllines

To test the detection performance of MixHMM on real tumor samples with known proportion of ‘FM’ cells, we used a dilution series of breast cancer cell lines studied by [12]. The genomic DNA from a cancer cellline (ATCC: CRL-2324D) was mixed with 0, 0.25, 0.5. 0.75, 1 proportion of DNA from a normal cellline (ATCC: CRL-2325D) and hybridized to Illumina Human109K BeadChips. A CNV detetion of the ‘normal’ cell line suggests that chromosome 6 and chromosome 16 harbor large regions of heterozygous deletion, so these two chromosomes are excluded in the following analysis. After the estimation of the BAF value of ‘A’ genotype in each sample, we use Equ. 11 to estimate the proportion of normal cells. We obtained 0, 0.25, 0.66, 0.86, 1 respectively, which is close to the proportion of normal DNA decribed above. The slight overestimation probably stems from the observation that such a cancer cell harbors more DNA than a normal cell. For example, if equal numbers of such cancer cells and normal cells are mixed, the proportion of normal cells is 0.5, while the proportion of normal DNA is less than 0.5.

**Figure 4:**
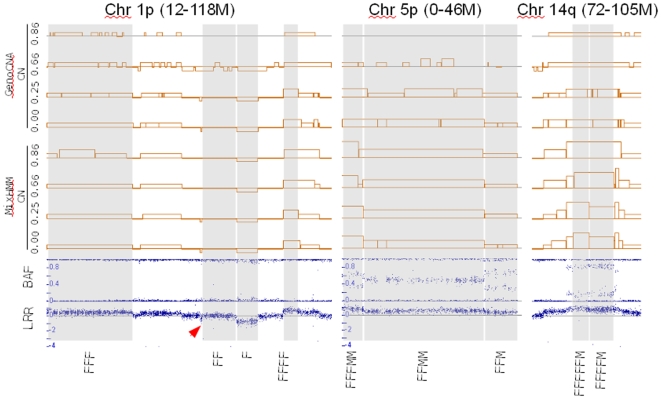
CN detection in dilution series of a breast cancer cell line (CRL-2324D). The numbers on the left of each track are the proportion of normal (‘FM’) cells, the BAF and LRR tracks are for pure tumor sample (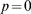). Some putative CNV states as detected with MixHMM from pure tumor sample are labeled below all tracks. The chromosome and approximate start and end location is labeled on top of each column. The arrow head in the left panel point to a short region with LRR values between those of 1n and 2n. In the CN tracks, the baseline (gray) represents 2n, and the copy numbers range from 0n through 7n.

We performed a CNV detection for each pure and mixed sample using a 20-state HMM. As detected from the (see Table S1) pure tumor sample, the breast cancer cellline has a very complex genotyping profile: the dominating regions are in LOH states instead of the nomal ‘FM’ state, and more than half (0.52) of the genome are amplified in various ways. In Figure 4, we show examples of the copy number results from samples mixed with different proportions of stromal cells. The left panel shows a long run of homozygosity (LOH regions) with different regions from chromosome 1p showing a variety of copy numbers. The middle panel shows three amplified regions (balanced and imbalanced) from chromosome 5p. The right panel includes a highly amplified region from chromosome 14q. The underlying truth about copy numbers in the cancer cellline is unavailable, yet the copy numbers detected from tumor samples mixed with 25%, 66% and 86% of normal cells are consistent with those from pure tumor sample. The copy numbers detected using GenoCNA are also shown in Figure 4. The MixHMM are more advantagous when the normal proportion is considerably high (greater than 50%).

**Figure 5:**
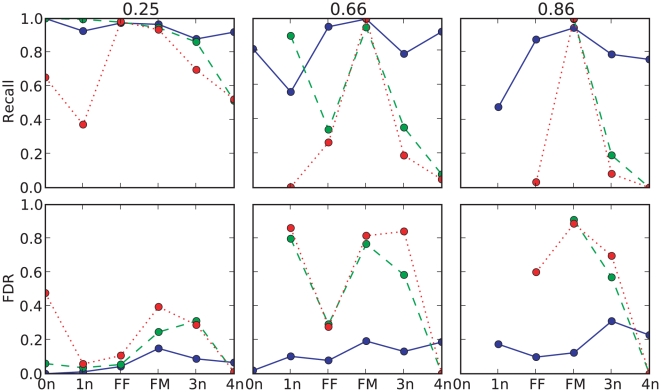
Comparison of three algorithms in dilution series of a breast cancer cell line (CRL-2324D). Each subplot shows the recovery (the upper row) and false discovery rates (the lower row) in a cancer sample with a certain proportion of normal cells (proportion labeled above each column). The collapsed CNV states are labeled on x-axis, with copy number = 0 (‘0n’), 1 (‘1n’), 2 (‘FF’,‘FM’), 3 (‘3n’), > = 4 (‘4n’). The blue points (connected with blue solid lines) are results using MixHMM, the red points (connected with red dotted lines) are for PennCNV and the green points (connected with green dashed lines) are for GenoCNA. When there are no SNPs detected in a state, there will be no point in the plot.

For comparison with other algorithms (PennCNV and GenoCNA) quantitively, we collapse the detected CNV states into six states used by PennCNV, and calculated the recovery and false discovery rates (FDRs) using the detection from the pure cancer cellline as reference. The results are shown in Figure 5. When mixed with a small proportion of normal cells (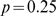), the performance of GenoCNA is comparable with MixHMM except for its low recovery (0.46) of states with more than three copy numbers. When mixed with a larger proportion of normal cells (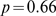), however, MixHMM has a much better performance. Note that the recovery of 1n (‘F’) state are higher for GenoCNA but it has a very high FDR too (0.79). Considering the genomic complexity of the cell line and the low density of the Human109K BeadChips, the detection results using MixHMM in samples mixed with up to 66% of stromal cells are satisfactory. The recovery rate for the 1n (‘F’) state in the sample with 66% stroma (0.56) is not as good as expected, because about half of regions detected as ‘F’ in pure tumor have a considerably higher median LRR value (an example of such a region is indicated with an arrow head in Figure 4). A possible explanation is that this ‘pure’ tumor sample is actually a mixture of two different clones, and their CNV states in the troublesome regions are different (for example, one is in ‘F’ and the other is in ‘FF’).

### Analysis of tumor samples

**Figure 6:**
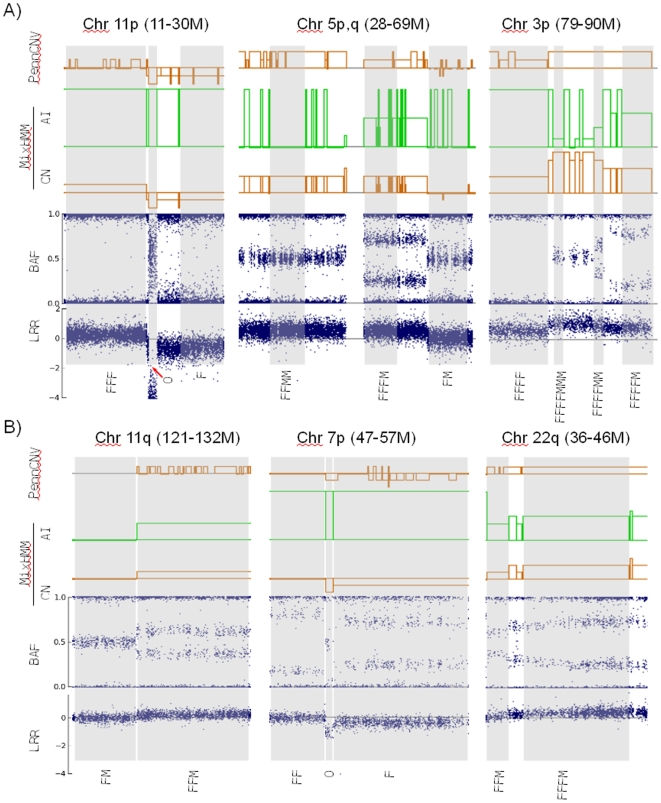
Detection of copy number (CN) and allelic imbalance (AI) in tumor samples. A) A melanoma sample (‘LAC-mel’) composed of almost pure tumor cells. B) A breast cancer sample (‘BT5’) with about 30% of normal cells. Choice state regions as detected by MixHMM are labeled below all tracks.. The top panels are results of PennCNV detection. On top of each panel we show the chromosome arm and approxiate start and end positions. The range of copy number (CN) is from 0 to 7 with the baseline represent 2n. The range of allelic imbalance (AI) is from 0 (for balanced states) to 0.5 (for LOH states), the AI of total deletion (‘O’) is set to 0.5 in this analysis. In the PennCNV track, the solid organge fragments on baseline represent copy-neutral LOH (‘FF’).

We have also applied our MixHMM algorithm with real tumor samples, both pure tumor samples and tumor samples ‘contaminated’ with stromal cells. In a melanoma pure tumor sample (‘LAC_mel’, unpublished data from the Halaban Lab) hybridized on Illumina's Human1M BeadChip, we have identified typical regions in each of the nine states for copy number up to 4 and some highly amplified regions (CN>4) (see Table S1 for a summary). In Figure 6A, we show examples of some detected regions compared with results of PennCNV. The left panel shows regions of total deletion (‘O’), one-copy deletion (‘F’), and three-copy LOH (‘FFF’) from chromosome 11p. The middle panel, from chromosome 5, shows a region of ‘normal’ state (‘FM’) and regions of two different four-copy heterozygous states: balanced (‘FFMM’) and imbalanced (‘FFFM’). The right panel, from chromosome 3p, shows a region of four-copy LOH (‘FFFF’) and regions of highly amplified states (CN = 5, 6, 7). Although the underlying truth about the copy number and allelic imbalance are unavailable, the assignments by MixHMM are consistent with manual annotation by comparing with the expected LRR and BAF patterns. In comparison with PennCNV, MixHMM detects more states. Not only can it detect states with higher copy numbers (up to 7), but different states with the same copy number can be distinguished by allelic imbalance. For example, LOH states with high copy numbers can be detected, which can be biologically important. MixHMM detection is also more accurate because of the more comprehensive state definitions. For example, some of the ‘FFF’ regions are misassigned as ‘FF’ and some of the ‘FFFM’ regions are misassigned as 3n by PennCNV.

Breast cancer biopsy samples are rarely pure unless they have been microdisssected. Here we use the published ‘BT5’ breast cancer data [20] to demonstrate the power of MixHMM. Following the procedure described in the methods, we estimate the proportion of normal cells in this sample (Figure S2) to be about 30%. In Figure 6B, we show the detection results using both MixHMM and PennCNV. As expected, the CNV detection using PennCNV in this heterogeneous dataset tend to be inaccurate. For example, it tends to assign one copy deletion (‘F’ in first column) as copy-neutral LOH, to assign 4n as 3n (‘FFFM’ in the last column). MixHMM, however, detects copy number and allelic imbalance in the cancer cells accurately (consistent with model and manual annotation)., in spite of the considerable contamination of stromal cells.

## Discussion

High throughout SNP-based genotyping arrays have been increasingly used to identify copy number variation and copy-neutral loss of heterozygosity, and have provided invaluable insight into the complexity of genomic variations, especially for disease related variations. The accuracy and density of genotyping arrays have improved rapidly, with current versions having a density of over one million SNPs/probes. However, new detection algorithms are needed to extract more detailed information about genome complexity from these genotyping data. And new algorithms are also needed to detect the genome complexity in tumor samples mixed with stromal cells, which is almost unavoidable in biopsy samples. Under the assumption that all the CNV events originate from the underlying normal state, here we present MixHMM, a novel HMM based algorithm, which can detect copy number, allelic imbalance and genotype accurately, from homogeneous samples or heterogeneous samples with tumor cells mixed with a certain proportion of stromal cells. We validated the technique using both simulation data and real tumor data including breast cancer and melanoma.

Allelic imbalance revealed by the genotyping data includes not only classical single copy LOH and copy-neutral LOH but, in principle, can include other forms of imbalance such as high-copy LOH and imbalanced amplification. Such information has not typically been a focus of whole genome analyses, but may provide insight into differing mechanisms of amplification at specific loci or mechanisms differing among individual patients. Our preliminary analyses suggest such events do occur in tumors. Only algorithms which can utilize the available data to detect these events will be able to identify how prevalent such changes are and lead to determining their functional significance. MixHMM models multiple states for a high-copy region, for example, three states instead one are used for a 4-copy region (see Figure 1 and Table 1). It is not only more genetically meaningful but also allows detection of all forms of allelic imbalance. Still another benefit of this modeling strategy is that we can assign a more meaningful genotype to each SNP, for example, instead of using ‘AB’ for a 4-copy heterozygous genotype, we distinguish ‘AABB’ or ‘ABBB’ or ‘AAAB’ instead.

Similarly to other HMMs for copy number analysis, such as wuHMM [21], MixHMM requires no training data. The six model parameters for each hidden state (mean and SD of LRR, mean and SD of 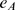 BAF, characteristic length of regions, proportion of SNPs) are provided with the package and can be easily modified by the users to adapt to special samples. We found that the CNV detection is robust to the transition parameters but is sensitive to the emission parameters (distributions of LRR and BAF).

Mismatches between data and model may cause inaccurate state assignments. These mismatches can stem from three different sources. The first type, which is the most common, stems from the fact that normalization procedures for the original density data were developed primarily for normal samples. In cancer samples with complex CNV events, BAF and LRR values of suboptimal quality are commonly found. The suboptimal quality can be manifested as asymmetric heterozygous BAF bands, characteristic LRR values for 2n considerably shifted from 0, genomic wave effects in LRR values, etc., none of which are biologically. In these cases, alternative normalization and preprocessing tools should be applied before CNV detection (see method 4.7). The second type of mismatch stems from a violation of our assumption that some regions of the ‘contaminated’ stromal genome are not normal, for example, in ‘F’ (one-copy deletion) state instead of ‘FM’ state, as from for instance, inherited copy number variants. In this case, the genotyping data from a paired stromal sample is needed for accurate CNV detection. The third type of data-model mismatch stems from the fact that the genome of tumor cells are sometimes not homogeneous (i.e. cancer cells with different copy number changes mix with each together), and this violates the model assumption that the input data are from a mixture of two kinds of genomes (see Figure 4 for an example). In this case, there will be different apparent proportions of normal cells in different regions, and small regions with alternating CNA states tend to be detected, which can be considered as a signal of inaccurate detection. Our model is not intended to distinguish among multiple clones because the state and proportion of tumor component cannot always be uniquely determined from the genotyping data of the mixed sample. For example a mixture with 50% ‘FFFM’ and 50% ‘FM’ gives BAF and LRR distributions exactly the same as those from 100% ‘FFM’ (germline CNV). Instead, we use the estimated global proportion (corresponding to the dominant clone of tumor cells) for CNV detection. Multiple regions of a tumor could be analyzed to more accurately deal with heterogeneous tumors [22].

Very recently, Sun et al. [19] have developed GenoCNA to detect the cancer CNV in a tumor samples contaminated with stroma. We have shown, using simulated samples and dilution series of cancer celllines, that MixHMM is significantly more accurate in detecting CNV in samples with a considerable proportion of stroma. In addition, CNV regions with copy number up to 7 can be detected effectively with the 20-state MixHMM model. Although detection of higher copy number will inevitably be less accurate because of the saturation effects in both hybridization and scanning, it is essential to detect the highly amplified regions in some cancer samples. For example, detection of patterns of high level amplification, termed ‘firestorms’ reported in many breast cancer samples [6], may be relevant for classification and prognostic significance.

MixHMM is designed to detect CNV states using BAF and LRR values, which are the typical output of Illumina BeadStudio. For other SNP array platforms such as the Affymetrix chip, the original outputs need to be transformed to BAF and LRR values beforehand. Fortunately, there are tools available for such tasks. For example, the PennCNV site (http://www.openbioinformatics.org /PennCNV) provides a protocol for that transformation. Although MixHMM currently only works for CNV detection from autosomes, it can be extended to cope with X, Y if the LRR values are well normalized.

In conclusion, our novel algorithm offers several distinct advantages over previous algorithms. MixHMM allows detection of copy number variations in tumor cells from a heterozygous sample contaminated with stromal cells, and it allows detection of higher copy numbers and richer allelic imbalance. MixHMM requires no training data, and the model can be easily adapted to special set of samples. These features are critical components of algorithms which will fully exploit the potential of the rapid evolving genotyping platforms for the detection of genomic variances and biomarkers.

## Methods

### Overview of the Model used by MixHMM

The CNV states listed in Table 1 are used as the hidden states in the model (20 hidden states for copy numbers up to 7). The initial/static state distributions (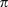) are estimated empirically. The state transition matrix (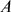) is not assumed to be stationary, but is estimated as a function of the distance between two SNP loci (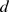) using Equ. 2. The emission probability (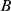) of an observation given a state is calculated as a combination of emission probabilities of both BAF signal and LRR signal using Equ. 3 and 4. For a pure sample, the normal distributions for LRR and BAF are estimated empirically. For a mixed sample, the proportion of stromal cells (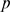) are estimated using Equ. 11, and the normal distributions for LRR and BAF are updated Equ. 6, 7, 9 and 10.

The Viterbi algorithm is used to decode the hidden state for each SNP, which are consequently converted into CNV regions. The copy number (CN) and allelic imbalance are then calculated from the state name (composed of ‘F’s and ‘M’s) of each CNV region (CN = #F+#M, AI = 0.5−#M/CN). To view the data and result in IGB browser (HTTP://igb.bioviz.org), SGR files are generated from BAF and LRR, and WIG files are generated from copy number and allelic imbalance. Genotype for each SNP can be optionally called after the state assignment: the genotype (one of four) with the greatest probability density at the BAF value. The population frequencies of ‘B’ allele (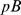) are optional (it is only important for accurate LOH detection), and we adapted them from the files in the PennCNV package [16]. To detect CNVs with a different model, just create a new model file using the provided model file ‘FM20_0.hmm’ (using more or less states and/or different model parameters). The time performance of the algorithm is insensitive to the number of states used.

### State transition probabilities

SNP loci are not evenly distributed in a chromosome. When two SNPs are closely located, the state of one SNP may be dependent on the other. However, as the distance becomes larger, the correlation will become weaker. When two SNPs are far apart, their states would be nearly independent. Here we use an exponential function to approximate the transition probabilities that have the above spatial property. Suppose the distance, measured by the number of nucleotides, between two adjacent SNPs is *d*. Let 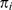 denote the probability of the stationary distribution of state 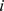, i.e., the proportion of SNPs in a state 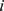. And let 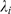 be the average length of regions in state 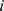. Define the transition probability from state 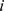 to a state other than 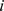 as
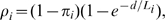
(1)where 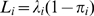. This definition assumes that the lengths of regions in state *i* and in states other than i have means equal to 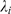 and 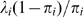, respectively. It has the following properties:
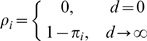
Here both 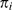 and 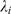 can be estimated empirically from the data. For simplicity, if there is a state change, we assume the next state is independent of current state (this can also be derived from the assumption and each CNV state originated from the ‘FM’ state). Therefore, the transition matrix 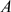 between two hidden states 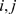 is given by:
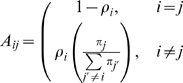
(2)


### Observation emission probabilities

Similar to PennCNV [16] and QuantiSNP [15], LRR and BAF are assumed to be independent for estimation of emission probabilities.

For LRR emission probabilities (the probability of observing a LRR value 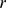 given a state 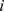), following Wang et al. [16], we also use a mixture of Gaussian and uniform distributions to reflect the effect of fluctuation (caused by genotyping error) in experiments
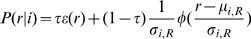
(3)where 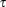 is the probability that a fluctuation happens, 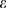 is the p.d.f. of a uniform distribution defined on all possible LRR values, and 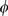 is the p.d.f. of the standard normal distribution. 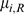,
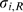
are the mean and standard deviation (SD) of LRR values in state 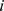. Note that different states with the same copy number share the same LRR distribution.

Similarly, emission probability of BAF given a state is modeled as a mixture of a uniform distribution and four normal distributions, each of which corresponds to one of the four genotype classes in Table 1. Recall that we denote the genotype classes as 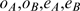 according the underlying germline genotypes. Assuming that each state comes from the ‘FM’ state, the four genotype classes must be derived from genotype AA, BB, AB, AB respectively (see Table 1). So the probability of observing each genotype class for a given tumor CNV state is the same as that of each germline genotype, which is 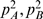,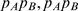, respectively, where 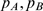 means the population frequency of ‘A’ allele and ‘B’ allele (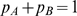). So we calculated the BAF emission probability (the probability of observing a BAF value 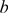 given a state 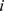) as
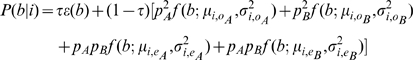
(4)where
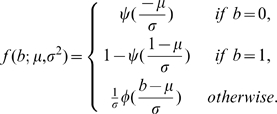
Here 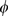 and 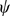 are the p.d.f. and c.d.f. of the standard normal distribution., and 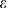 is the p.d.f. of a uniform distribution on all possible BAF values. 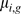,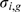 are the mean and SD of the BAF values of genotype class 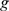, in state 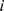.

**Figure 7:**
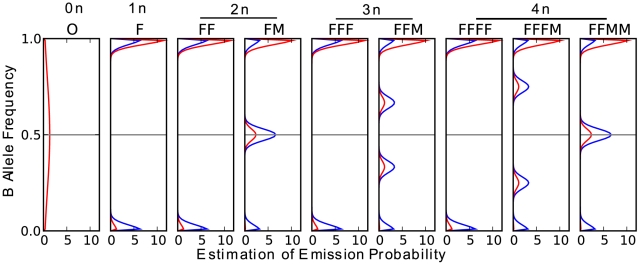
BAF emission probability. The blue lines represent the distributions when 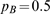, the red lines represent the distributions when 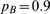. Here 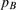 stands for the population frequency of ‘B’ allele. Each subplot represent the distributions of a certain CNV state (state names labeled on top). The first track on top of the graphs are copy numbers of each state.

Figure 7 shows the BAF emission probability distribution for all the nine states with copy number up to 4. The value of 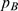 can significantly influence the distinction of a LOH state, in that it can give the two homozygous genotypes (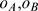) very different implications. For example, when 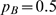, the probabilities of observing 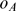 and 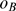 are the same (0.25 for ‘FM’ state and 0.5 for ‘FF’ state); however when 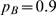, the probabilities of observing 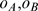 are very different (0.01,0.81 for ‘FM’ and 0.1, 0.9 for ‘FF’). Thus, LOH states will be much more distinguishable from other states when 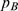 is far from 0.5 and the minority allele is observed.

### Estimation of model parameters for MixHMM

We provide a 20-state model for copy numbers from 0 through 7, as listed in Table 1. The 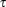 in Equ. 3 and 4 is platform specific, and is set to be 0.01 for Illumina SNP BeadArray. The average length of regions and proportion of SNPs in each state (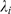 and 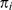 in Equ. 1) are sample specific. We set them empirically based on manually annotated breast cancer data and found that the CNV detection of MixHMM is relatively robust to these parameters (data not shown).

In a homogeneous sample, the normal distributions of LRR and BAF are set as follows. For the homozygous deletion state (‘O’), the mean of LRR and BAF are set to be −4 and 0.5, repectively. For another state, the mean of the LRR is calculated from the copy number using the equation provided by [23] and the mean of the 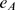 BAF is set to be the MCP in Table 1. The standard deviation of LRR and 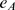 BAF of a state are determined empirically using manually annotated cancer data. The normal distributions of BAF for other genotype classes are calculated from that for 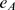: the distribution for 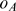 is set to be that for 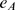 of the LOH state with the same copy number; the distributions of 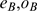 is calculated from those of 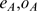 considering the symmetric property of BAF distribution.

In a mixed sample with a proportion (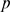) of stromal cells, we update the normal distributions of LRR and BAF with 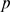 as described in the following two sections.

### Calculation of normal distributions of LRR in a mixed sample

We can derive the formulation for the R score in a tumor sample mixed with a proportion (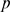) of normal cells with an assumption of linearity:
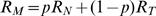
(5)where 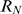 and 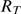 are the R scores contributed from the normal and tumor DNAs in the mixed sample, and both of them follow log-normal distributions. It can be shown that approximately the log ratio of 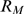 also follows a normal distribution [24], of which the parameters can be estimated by:
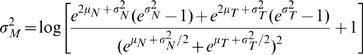
(6)

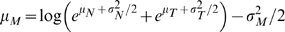
(7)where
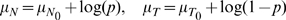


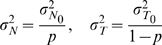
Here 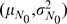 and 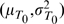 are the parameters of the normal distributions for LRR scores in pure normal and pure tumor samples, respectively. Specifically, when 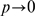,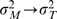, we have 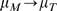 asymptotically.

### Calculation of normal distributions of BAF in a mixed sample

Assuming that a CNV region in tumor cells is originated from the corresponding region in the stromal cells, a mixed ‘genotype’ must derives from the mixture of genotypes of the same class. Based on the model of BAF described by Nancarrow et al. [23], we can derive the mixed BAF of a given genotype class 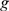 (one of 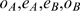 as described before) as a linear combination of BAFs contributed by normal and tumor cells with the same genotype class as below:
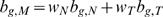
(8)where 
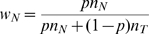
, 
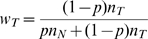
, 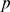 is the proportion of normal cells, 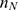 and 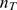 are the copy number in normal and tumor cells. 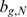 and 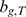 are BAF signals contributed from the normal and tumor DNA. Thus 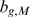 follows a normal distribution, of which the parameters can be estimated by:
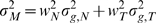
(9)

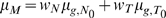
(10)where
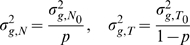
Here 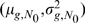 and 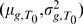 are the parameters of the normal distributions of BAF scores for genotype 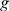 in pure normal and pure tumor samples, respectively.

### Data preprocessing for tumor samples

The array designs and sample descriptions for the tumor datasets can be found in the results section. For tumor samples, the BAF and LRR directly exported from Illumina BeadStudio may be problematic: the BAF are often asymmetric and the mean LRR for diploid (CN = 2) are sometimes shifted considerably from 0. So, we use the follow protocol for data preprocessing.

First, export X, Y from BeadStudio. Second, use tQN [20] to adjust the asymmetric BAF bands. Next, genomic waves reflected in the LRR values are (optionally) reduced by the GC regression model [25] included in the PennCNV package.

### Estimation of the proportion of normal cells and LRR shift

In order to estimate the proportion of normal cells in a mixed sample, it is necessary to also adjust the LRR baseline if there is a genome-wide LRR value shift (an upstream normalization error leading to LRR value zero not mapping to copy number 2). We provide a plotting tool to generate a genome-wide view of BAF and LRR (Figure S2). First, detect the lowest consistent bands in the LRR plot (ignore the total deletion regions as they are short and rare, thus not consistent enough to be used.), and decide whether they are from one copy or two copy by checking the relevant BAF pattern: some of the two-copy regions (FM state) have an obvious BAF band at 0.5, while others (FF) with the same LRR will have no BAF band at 0.5. On the other hand, there will be no one-copy regions (F) which have a BAF band at 0.5. If the lowest band is one copy, the second lowest band corresponds to two copy. Use the estimated median LRR value of 2-copy band for ‘LRR_baseline’ value. All the LRR values are shifted according to the ‘LRR baseline’ annotated above.

To estimate the proportion of normal cells (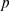) in a mixed sample (and in which the LRR shift has been corrected), we detect the ‘A’ band in the F regions or the ‘AA’ band in the FF regions (identified as described above) in the BAF plots. There will be no band between 0 and 0.5 in a pure tumor sample and a single band up to 0.45 for tumor samples mixed with normal host cells. We annotate the estimated median value of this single band (*b*) and apply the following formula derived from SiDCoN [23] to calculate 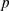:
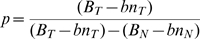
(11)where 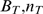 is the B allele copy number and total copy number of the tumor derived from the given genotype (‘A’ or ‘AA’) annotated above; *b* is the annotated BAF value of the given genotype; 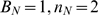 is the B allele copy number and total copy number of the normal genotype (‘AB’). In the uncommon case that no F or FF states are identified in the sample, higher copy states can also be used with the same formula.

### Simulation of a sample with MixHMM

To simulate a sample, we use the actual SNP locations and population B allele frequency of Illumina Human550K. The 20-state HMM represent states with copy numbers up to 7 is used for simulation. We simulate a 100-SNP or 300-SNP region for each CNV state on each chromosome. For each SNP, we sample randomly from the BAF and LRR emission distributions of the simulated state. Then the CNV state is detected using the same HMM. To evaluate the performance of PennCNV on the simulated sample, we use a 6-state HMM model (in PennCNV format) collapsed from the 20-state HMM.

### Detection of CNV states with other algorithms

For PennCNV [16], the 2008Nov19 version is used. PennCNV default options to adjust the LRR and BAF are turned off (“–nomedianadjust –nobafadjust –nosdadjust”), as the values are already normalized in the preprocess steps. The “–loh” option is enabled to compare with the MixHMM results.

The genoCNA function in the genoCN R package [19] is used with default parameters.

## Supporting Information

Figure S1 Recovery rates of copy number and allelic imbalance from the simulated 300-SNP regions. The numbers on the left side are proportions of ‘FM’ cells. Values of each copy number/allele imbalance comes from the simulations of 220 regions.
(0.19 MB EPS)

Figure S2 Estimation of the proportion of normal cells. This is part of the BAF-LRR plot generated from the BT5 breast cancer sample (chromosome 5 to 12). The chromosome numbers are labeled below each track. A SNP is represented as a point in each track. The range of BAF is 0 through 1, the range of LRR is -4 through 2. The highlighted (red lines) genotype is ‘A’ in state ‘F’ and the estimated BAF value is 0.25, so the *p* is calculated to be 0.33 using Equ. 11.
(0.38 MB TIF)

Table S1 Summary of CNV states detected in tumor samples using MixHMM.
(0.03 MB DOC)
