# Supplementary figures and images for: MixHMM: Inferring Copy Number Variation and Allelic Imbalance Using SNP Arrays and Tumor Samples Mixed with Stromal Cells
Source: PLoS One. 2010 Jun 1;5(6):e10909. doi: 10.1371/journal.pone.0010909 (PMC2879364; doi:10.1371/journal.pone.0010909)

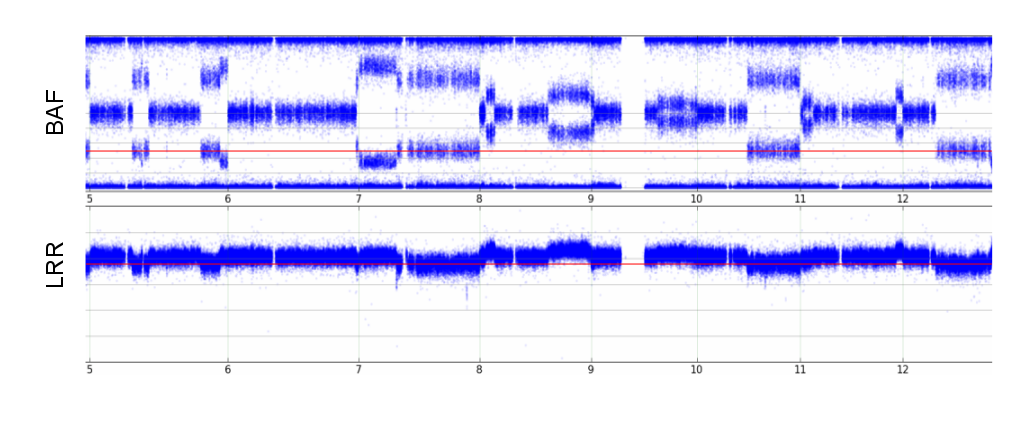

Supplement: Figure S2 — Estimation of the proportion of normal cells. This is part of the BAF-LRR plot generated from the BT5 breast cancer sample (chromosome 5 to 12). The chromosome numbers are labeled below each track. A SNP is represented as a point in each track. The range of BAF is 0 through 1, the range of LRR is -4 through 2. The highlighted (red lines) genotype is ‘A’ in state ‘F’ and the estimated BAF value is 0.25, so the p is calculated to be 0.33 using Equ. 11. (0.38 MB TIF) [file pone.0010909.s002.tif]
