# Supplementary material for: MixHMM: Inferring Copy Number Variation and Allelic Imbalance Using SNP Arrays and Tumor Samples Mixed with Stromal Cells
Source: PLoS One. 2010 Jun 1;5(6):e10909. doi: 10.1371/journal.pone.0010909 (PMC2879364; doi:10.1371/journal.pone.0010909)
Supplement: Table S1 — Summary of CNV states detected in tumor samples using MixHMM. (0.03 MB DOC) [file pone.0010909.s003.doc]

Table S1: Summary of CNV states detected in tumor samples using MixHMM.

Table S1: Comparison of copy number recovery rates with PennCNV in simulated 300-SNP regions

The recovery rates (the cell values) are calculated from a simulation of 20 states on chromosome 1. Each state is simulated as a 300-SNP region. To compare with the PennCNV assignments, the 20 states, which represent CNV states for copy numbers up to 7, are collapsed into 6 states defined in PennCNV. *p* means proportion of normal stromal cells mixed in the simulated sample.
